# Greek Version of the mHealth App Usability Questionnaire (GR-MAUQ): Translation and Validation Study

**DOI:** 10.3390/healthcare13243285

**Published:** 2025-12-15

**Authors:** Ioannis Kouroutzis, Pavlos Sarafis, Maria Malliarou

**Affiliations:** Department of Nursing, University of Thessaly, 41500 Larissa, Greecemalliarou@uth.gr (M.M.)

**Keywords:** MAUQ, validation, mHealth, questionnaire, usability, application, mobile health

## Abstract

**Background/Objectives**: The mHealth App Usability Questionnaire (MAUQ) is a validated instrument specifically designed to evaluate the usability of mobile health applications. The aim of this cross-sectional study, was to translate, culturally adapt, and validate the Greek version of the MAUQ. **Methods**: The 21-item mHealth App Usability Questionnaire (patient, interactive version) was forward- and back-translated from English into Greek, following scientific guidelines for translation and cross-cultural adaptation. Exploratory factor analysis was carried out to evaluate construct validity, disclose underlying structures and reduce the number of variables in MAUQ. Principal component analysis (PCA) was chosen as extraction method using Varimax rotation. **Results**: The study sample included 385 participants, of whom 66.0% were women, with a mean age of 42.2 years (SD = 14.0). The factor analysis yielded three components that together accounted for 67.8% of the total variance. The “system information arrangement” factor comprised six items and explained 24.9% of the variance. The “usefulness” factor contained seven items and contributed 23.7%, while the “ease of use and satisfaction” factor included eight items and accounted for 19.2%. All items showed satisfactory corrected item–total correlations, exceeding 0.30. Cronbach’s alpha values were 0.92 for “ease of use and satisfaction,” 0.89 for “system information arrangement,” and 0.92 for “usefulness,” demonstrating strong reliability for each subscale. Overall, total’s scale reliability was a = 0.93. **Conclusions**: The Greek version of the mHealth App Usability Questionnaire (GR-MAUQ), demonstrated strong psychometric properties and confirm its suitability for assessing the usability of mHealth applications among Greek-speaking users.

## 1. Introduction

Mobile health (mHealth) applications have become an integral part of modern healthcare delivery, supporting functions such as remote monitoring, disease self-management, health data collection, patient education and behavioural interventions [1,2,3]. Their increasing use is driven by the widespread adoption of smartphones and the shift towards digitally supported, patient-centred care models [4]. When designed and implemented properly, mHealth applications can improve clinical outcomes, enhance patient engagement and reduce healthcare costs [5,6]. However, poor usability remains one of the most common reasons for app abandonment, along with hidden costs, data protection concerns and loss of interest [7]. As usability directly affects user satisfaction, engagement, and long-term adoption of the application, its systematic evaluation is considered essential before large-scale deployment [8].

Usability assessment of mHealth applications is most often conducted through standardized questionnaires due to their practicality, reproducibility, and suitability for large samples [9]. However, widely used tools, such as the System Usability Scale (SUS) and the Post-Study System Usability Questionnaire (PSSUQ), were originally developed for general software systems and are not sensitive to usability dimensions specific to mobile health technologies [10,11]. To address this gap, Zhou et al. developed the mHealth Application Usability Questionnaire (MAUQ), a validated tool specifically tailored to the characteristics of mHealth applications and their intended users [12]. The MAUQ includes four versions based on the type of application (interactive vs. stand-alone) and the user group (patients vs. healthcare providers) and has demonstrated strong psychometric properties in the original English version [12].

Since its development, the MAUQ has been successfully translated and validated in multiple languages, including Italian, German, Spanish, Chinese, Malay, Persian and Canadian French, confirming its reliability and cultural adaptability in diverse clinical populations [13,14,15,16,17,18,19,20]. However, there is currently no validated Greek version of the MAUQ, despite the increasing adoption of digital health solutions in Greece, both in clinical practice and in public health initiatives. The absence of a culturally adapted Greek tool limits the ability of researchers, clinicians, and developers to systematically measure and compare usability outcomes in Greek-speaking populations.

Therefore, the aim of this study was to translate, culturally adapt, and validate the Greek version of the MAUQ, which will enable standardized usability assessment in future mobile health research and support the development and implementation of evidence-based digital health interventions in Greece.

## 2. Materials and Methods

### 2.1. Overview of the MAUQ

The mHealth App Usability Questionnaire (MAUQ) was originally developed by Zhou et al. as a validated instrument specifically designed to evaluate the usability of mobile health applications [12]. The MAUQ provides four versions based on target user (patients or healthcare providers) and app interaction type (interactive or standalone), allowing usability assessment. In this study, the patient version for interactive mHealth apps was used. This version consists of 21 items, distributed across three subscales: (a) Ease of Use and satisfaction—8 items, (b) system information arrangement—6 items (c) Usefulness—7 items. In the original validation, the MAUQ demonstrated strong internal consistency, with Cronbach’s α values of 0.895 (Ease of Use and satisfaction), 0.829 (system information arrangement), and 0.900 (Usefulness) [12]. All items are rated on a 7-point Likert scale ranging from 1 (strongly disagree) to 7 (strongly agree), with higher scores reflecting better usability perceptions.

### 2.2. Procedure

Permission to translate and validate the original English version of the mHealth App Usability Questionnaire (MAUQ) into Greek for research purposes was obtained from one of the authors (Leming Zhou).

The mHealth App Usability Questionnaire (MAUQ) was initially translated into Greek independently by two researchers MM and IK, who were fluent in English. The two drafts were then compared item by item until the consensus was reached through discussion by an expert panel that included P.S., M.M. and I.K. all experienced in questionnaire development. The revised draft of the Greek version of the mHealth App Usability Questionnaire (Gr-MAUQ) that was agreed upon was then translated back into English by two bilingual individuals who had no prior knowledge of the instrument. The back translations were compared, and inconsistencies were addressed until a final back-translation document was agreed upon. This version was then compared with the original English version of the mHealth App Usability Questionnaire (MAUQ) for final confirmation of the linguistic accuracy. The backward translation was also checked after a pilot test of the questionnaire in Greek in a sample of 10 people, in order to capture problems regarding the wording and comprehension of the questions (if they did not understand a word, if a word or expression was offensive or unacceptable, etc.). From the pilot study, no problems arose. This resulted in the final version of the Greek mHealth App Usability Questionnaire (GR-MAUQ).

### 2.3. Study Design

A cross-sectional study was conducted to achieve the aim of the research. The sample was obtained through snowball sampling, a widely used non-probability method in which the researchers initially recruited a small number of participants who met the inclusion criteria and agreed to take part in the study. These participants subsequently referred other eligible individuals, who in turn recommended additional participants, creating a referral chain until the target sample size was reached. The target population consisted of adult citizens of both sexes, able to understand written and spoken Greek, capable of comprehending the study protocol, and with access to an Internet-connected electronic device (computer, tablet, or smartphone). Data collection took place between October and December 2024. The minimum required sample size was calculated as 385 individuals using the Raosoft Sample Size Calculator, with a 5% margin of error, 95% confidence level, total population size of 10,413,982, and response distribution of 50% [21].

Data were collected through an electronic questionnaire created using Google Forms, a free and user-friendly platform that does not require specialized technical skills. The form consisted of three sections: an information and consent form; socio-demographic questions; and the Greek version of mHealth App Usability Questionnaire (GR-MAUQ). All participants were reassured that their anonymity and confidentiality would be protected without obtaining any personal, identifying information. They have primarily been informed about all the details of the study (scope, their right to withdraw).

### 2.4. Statistical Analysis

Quantitative data were summarized as means with standard deviations or as medians with interquartile ranges, whereas qualitative data were presented as counts and percentages. To assess construct validity, identify latent dimensions, and reduce the total number of MAUQ variables, an exploratory factor analysis was performed. Principal component analysis (PCA) with Varimax rotation served as the extraction technique, and sampling adequacy was evaluated using the Kaiser–Meyer–Olkin (KMO) statistic. Factor retention was based on eigenvalues that were exceeding 1.00 and scree plot inspection. For item retention, factor loadings of 0.40 or higher were considered acceptable. Internal consistency was examined through Cronbach’s alpha, with values ≥0.70 deemed satisfactory. Relationships among variables were examined using Spearman’s rho. For discriminant validity, associations between MAUQ factors and gender were assessed with the Mann–Whitney test. All *p*-values were two-sided, statistical significance was defined as *p* < 0.05, and analyses were performed using SPSS version 27.0.

## 3. Results

The sample consisted of 385 individuals (66.0% women), with mean age being 42.2 years (SD = 14.0 years), who were aware of the myHealth app, an application developed by e-Government Center for Social Security (IDIKA) organization of the Greek Ministry of Digital Governance. Their characteristics are presented in Table 1. The majority of the participants 79.5% lived in a city, 49.9% were MSc holders and 50.6% were married. Also, 85.4% of the sample rated their health as good/very good, while 24.9% had been diagnosed with a chronic disease. Advanced digital literacy had 58.2% of the sample and 30.6% were using a little this app.

Analytical description of MAUQ items is provided in Table 2. The percentages of agreement ranged from 49.9% to 92.9%. Specifically, 49.9% of participants agreed with the statement “I19. Using the app, I had many more opportunities to interact with my health care provider” and 52.9% with the statement “I20. I felt confident that any information I sent to my provider using the app would be received.” Also, 92.9% of participants agreed with the statement “I7. I would use this app again.” and 89.5% with the statement “I2. It was easy for me to learn to use the app”.

An exploratory factor analysis was performed using the Principal Components approach with Varimax rotation, resulting in a three-factor solution, as shown in Table 3. The KMO statistic was 0.95, and Bartlett’s test indicated adequate factorability (*p* < 0.001). All items loaded above 0.40 on their respective factors, and no notable cross-loadings were observed. Thus, no items were removed from the scale. Together, the three factors accounted for 67.8% of the total variance. The first factor, labeled “system information arrangement,” consisted of six items and contributed 24.9% of the variance. The second factor, “usefulness,” included seven items and explained 23.7% of the variance, while the third factor, “ease of use and satisfaction,” comprised eight items and accounted for 19.2% of the variance. The factors that emerged from this analysis were identical to factors proposed in the literature.

Table 4 presents the corrected item–total correlations and the Cronbach’s alpha values that would result if any item were removed from each of the three factors. All items demonstrated adequate item–total correlations, exceeding the 0.30 threshold [22]. The Cronbach’s alpha values were 0.92 for the “Ease of use and satisfaction” factor, 0.89 for “System information arrangement,” and 0.92 for “Usefulness,” confirming strong internal consistency for all subscales. Furthermore, deleting any item did not lead to an improvement in reliability, indicating that all items were appropriate to retain. The overall scale showed a reliability coefficient of α = 0.93.

Mean score was 5.66 (SD = 0.82) for factor “Ease of use and satisfaction”, 5.17 (SD = 0.91) for “System information arrangement”, 4.97 (SD = 1.02) for “Usefulness” and 5.29 (SD = 0.82) for total score (Table 5). There were significant positive correlations between all scores (*p* < 0.001).

MAUQ factor scores by gender are presented in Table 6. Women had significantly higher scores on the factors “System information arrangement”, “Usefulness” as well as in total score, indicating more positive views on the application. On the contrary, the score of “Ease of Use and Satisfaction” factor was similar in men and women.

## 4. Discussion

This study detailed the process of translating and validating the English version of mHealth App Usability Questionnaire (MAUQ) into the Greek language. No significant cultural or linguistic challenges were encountered during the adaptation process.

Data analysis proved the high reliability and validity of the GR-MAUQ. The values for Cronbach alpha for the entire questionnaire and for the three subscales: ease of use and satisfaction, system information arrangement, and usefulness, were high (0.93, 0.92, 0.89, and 0.92), indicating strong internal consistency. Exploratory factor analysis was carried out to evaluate construct validity of GR-MAUQ.

Exploratory factor analysis (EFA) provided strong evidence supporting the construct validity of the Greek version of the MAUQ. The Kaiser–Meyer–Olkin (KMO) sampling adequacy measure (0.95) indicated that the data were highly suitable for factor analysis, while the statistically significant Bartlett test of sphericity (*p* < 0.001) confirmed the presence of adequate inter-item correlations. The resulting three-factor structure is consistent with the theoretical model of the original instrument and the absence of cross-loadings, combined with factor loadings exceeding the minimum acceptable threshold of 0.40, indicates clear factor differentiation and item stability. Due to sample size limitations, we were not able to conduct a CFA on an independent dataset to confirm the three-factor structure. Future research with a larger sample is therefore recommended to perform CFA and further substantiate the psychometric robustness of the GR-MAUQ.

The three extracted factors jointly explained 67.8% of the total variance, a value considered satisfactory for psychometric instruments related to usability. Among them, the factor labeled “System Information arrangement” accounted for the highest percentage of variance (24.9%) and reflects the structural and organizational aspects of the application interface. The second factor, “Usefulness,” explained 23.7% of the variance and captures the perceived functional value of the application in supporting health-related tasks. The third factor, “Ease of Use and Satisfaction,” explained 19.2% of the variance and is related to the user experience component of interacting with the application, including perceived usability and overall satisfaction.

Factor analysis of Persian version of the MAUQ for interactive applications showed three factors as well. The variance interpretation rates of the three factors for interactive applications were 34.31% (User interface and satisfaction), 22.50% (Usefulness), and 14.65% (Easy to use), and the cumulative variance interpretation rate after rotation was 71.47%.

The distribution of variance among the factors demonstrates that the tool is consistent with previous validations in other languages. The close alignment between the Greek structure and the original conceptual model enhances the cross-cultural applicability of the MAUQ and supports its use as a reliable tool in Greek-speaking populations.

Similarly to our findings, the Italian [13], German [14], Spanish [15] and Persian [19] adaptations also retained a three-factor solution that explained between 60% and 70% of the total variance, which is comparable to the 67.8% observed in the Greek sample. The reliability indices of the Greek version were also aligned with international evidence: Cronbach’s α values ranged from 0.89 to 0.92 for the three subscales and 0.93 for the total scale, which is in line with the coefficients reported in the Italian (0.78–0.92), German (0.86–0.96), and Chinese (0.82–0.98) versions.

The relatively high usability scores observed in our study—especially for “Ease of use and satisfaction” (mean = 5.66)—are comparable to the German (mean = 5.07) study, where participants also demonstrated strong acceptance of the evaluated apps. Overall, the convergence of factor structure and psychometric indices across studies reinforces the validity of the MAUQ as a globally adaptable instrument.

The present study identified statistically significant gender differences in two of the three MAUQ dimensions, with women reporting higher scores on “System information arrangement,” “Usefulness,” and on the total MAUQ score, while no difference was observed for “Ease of use and satisfaction.” These findings suggest that although both men and women found the app easy to use, women perceived the app as more functionally valuable and better structured. Greek women are generally more active in seeking health information online because they are more engaged in managing health for them and their families. This attitude may contribute to their perception of the app as more useful. Previous surveys indicate that women in Greece are slightly more likely than men to use the internet for health purposes, which aligns with our finding of higher MAUQ scores among women. The gender differences observed in the present study are consistent with findings from the Italian [13] and Chinese [16] adaptation of the MAUQ while in contrast with the original validation by Zhou et al. [12], where no demographic variable, including gender, significantly influenced MAUQ scores. In our sample, women reported significantly higher ratings for “System information arrangement,” “Usefulness,” and the total MAUQ score, suggesting stronger perceived value and usability of the app. A different pattern was noted in the Chinese validation study [16], in which the sample was heavily dominated by women (90.4%) and the authors attributed the high internal consistency of the scale partly to the overrepresentation of female respondents. They concluded that women were more willing to engage in usability-based surveys and more inclined to use mHealth apps for health-related purposes—a finding also acknowledged in Zhou et al. [12]. We can also note that men and women may differ in how they perceive and use digital health tools due to distinct behavioral characteristics and social roles, which ultimately shape their expectations of the technology. This interpretation is supported by the Chinese validation study, where the overwhelmingly female sample (90.4%) was associated with high reliability indices and greater interest in usability evaluation, suggesting that women may not only be more actively engaged with mHealth applications, but also evaluate usability more critically and consistently.

Emerging evidence from the Greek, Italian, and Chinese versions suggests that gender neutrality may not be a universal feature of the instrument, but rather a consequence of sample characteristics or the cultural context in the original study. Overall, current cross-cultural validations indicate that women may exhibit higher perceived usefulness and stronger evaluation of interface structure, while ease of use tends to be rated similarly across genders. This pattern highlights the importance of examining gender not only as a background variable but also as a potential determinant of perceptions of mHealth usability, with implications for user-centered design, personalization strategies, and future validation research. Gender may play a more meaningful role in mHealth usability perceptions than originally assumed. While Zhou et al. suggested that MAUQ performance is demographically neutral, later studies show that sample composition, cultural expectations, health engagement patterns, and gender-based digital behavior can influence usability ratings. This implies that the absence of gender effects in the original study may be related more to the characteristics of the sample than to the inherent neutrality of the instrument.

## 5. Limitations

Although our study can present major strengths such as following internationally accepted procedures for cross-cultural adaptation, and assessment of psychometric properties in a sufficiently powered sample, some limitations should be acknowledged. First, the sample was obtained through snowball sampling and consisted mainly of highly educated participants with advanced digital literacy, which may limit the generalizability of the results to populations with lower educational or technological access levels. Future validation with probability-based or stratified sampling is recommended to capture more diverse digital literacy levels.

## 6. Conclusions

This study successfully translated, culturally adapted, and validated the Greek version of the mHealth App Usability Questionnaire (GR-MAUQ), demonstrating strong psychometric properties and confirming its suitability for assessing the usability of mHealth applications among Greek-speaking users. The three-factor structure identified—Ease of Use and Satisfaction, System Information Arrangement, and Usefulness—closely replicated the original conceptual model and aligned with previous international adaptations of the MAUQ. The three-factor structure confirmed the measurement model of the original MAUQ, indicating that the GR-MAUQ reflects the same underlying constructs. The GR-MAUQ represents a valid and reliable tool for use in future research, clinical practice, and digital health evaluation projects in Greece. Its availability in Greek contributes to the standardization of usability assessment and supports the broader adoption of evidence-based mHealth solutions in the national health system.

High internal consistency, well-explained variance, and clear factor loadings support the reliability and construct validity of the instrument. Furthermore, the identification of significant gender differences highlights the importance of considering user characteristics when interpreting usability results and reinforces the need for gender-responsive approaches to mHealth design and evaluation.

The GR-MAUQ represents a valid and reliable tool for use in future research, clinical, and digital health evaluation projects in Greece. Its availability in Greek contributes to the standardization of usability evaluation and supports the wider adoption of evidence-based mHealth solutions in the national healthcare system as it could help developers refine design, help clinicians assess patient use, and help policymakers make informed decisions on digital health tools’ adoption and implementation.

## Figures and Tables

**Table 1:** Sample characteristics.

	n (%)
Gender	
Men	131 (34.0)
Women	254 (66.0)
Age, mean (SD)	42.2 (14.0)
Place of residence	
Village (<2000 residents)	43 (11.2)
Small city (2000–10,000 residents)	36 (9.4)
City (>10,000 residents)	306 (79.5)
Educational level	
Primary school	0 (0)
Middle/High school	32 (8.3)
College	17 (4.4)
University	144 (37.4)
MSc	192 (49.9)
Married/Civil partnership	202 (52.4)
Health status	
Bad	3 (0.8)
Moderate	53 (13.8)
Good	196 (50.9)
Very good	133 (34.5)
Chronic disease	96 (24,9)
Digital literacy	
Beginner	3 (0.8)
Intermediate	102 (26.5)
Advanced	224 (58.2)
Professional/Expert user	56 (14.5)
Frequency of using the app	
Never	74 (19.2)
A little	118 (30.6)
Moderately	89 (23.1)
Often	80 (20.8)
Very often	24 (6.2)

**Table 2:** Description of MAUQ items.

	Totally Disagree	Disagree	Probably Disagree	Neither Agree Nor Disagree	Probably Agree	Agree	Totally Agree	Percentage of Agreement (%)
	n (%)	n (%)	n (%)	n (%)	n (%)	n (%)	n (%)	
I1. The app was easy to use.	2 (0.6)	1 (0.3)	4 (1.1)	36 (10.1)	81 (22.7)	173 (48.5)	60 (16.8)	88.0
I2. It was easy for me to learn to use the app.	1 (0.3)	0 (0)	4 (1.1)	32 (9.1)	50 (14.2)	184 (52.3)	81 (23)	89.5
I3. I like the interface of the app.	2 (0.6)	3 (0.8)	12 (3.4)	53 (15)	73 (20.7)	164 (46.5)	46 (13)	80.2
I4. The information in the app was well organized, so I could easily find the information I needed.	3 (0.9)	7 (2)	13 (3.7)	47 (13.4)	84 (23.9)	157 (44.6)	41 (11.6)	80.1
I5. I feel comfortable using this app in social settings.	4 (1.1)	5 (1.4)	12 (3.4)	59 (16.9)	64 (18.3)	154 (44)	52 (14.9)	77.1
I6. The amount of time involved in using this app has been fitting for me.	2 (0.6)	5 (1.4)	7 (2)	44 (12.5)	71 (20.1)	175 (49.6)	49 (13.9)	83.6
I7. I would use this app again.	1 (0.3)	0 (0)	0 (0)	24 (6.9)	48 (13.7)	175 (50)	102 (29.1)	92.9
I8. Overall, I am satisfied with this app.	3 (0.9)	1 (0.3)	5 (1.4)	34 (9.7)	62 (17.8)	179 (51.3)	65 (18.6)	87.7
I9. Whenever I made a mistake using the app, I could recover easily and quickly.	6 (1.7)	7 (2)	14 (4)	124 (35.2)	76 (21.6)	98 (27.8)	27 (7.7)	57.1
I10. This mHealth app provided an acceptable way to receive health care services.	4 (1.2)	2 (0.6)	11 (3.2)	50 (14.4)	82 (23.6)	159 (45.8)	39 (11.2)	80.7
I11. The app adequately acknowledged and provided information to let me know the progress of my action.	2 (0.6)	5 (1.4)	13 (3.7)	61 (17.6)	81 (23.3)	152 (43.8)	33 (9.5)	76.7
I12. The navigation was consistent when moving between screens.	3 (0.9)	6 (1.7)	13 (3.7)	69 (19.8)	79 (22.7)	154 (44.3)	24 (6.9)	73.9
I13. The interface of the app allowed me to use all the functions (such as entering information, responding to reminders, viewing information) offered by the app.	3 (0.9)	6 (1.7)	20 (5.7)	68 (19.5)	84 (24.1)	145 (41.7)	22 (6.3)	72.1
I14. This app has all the functions and capabilities I expect it to have.	6 (1.7)	18 (5.2)	24 (7)	79 (23)	92 (26.7)	107 (31.1)	18 (5.2)	63.1
I15. The app would be useful for my health and well-being.	2 (0.6)	5 (1.4)	6 (1.7)	40 (11.5)	74 (21.3)	168 (48.3)	53 (15.2)	84.8
I16. The app improved my access to health care services.	3 (0.9)	9 (2.6)	17 (4.9)	62 (18)	69 (20)	143 (41.4)	42 (12.2)	73.6
I17. The app helped me manage my health effectively.	5 (1.4)	12 (3.4)	23 (6.6)	98 (28)	72 (20.6)	107 (30.6)	33 (9.4)	60.6
I18. The app made it convenient for me to communicate with my health care provider.	9 (2.6)	16 (4.6)	24 (6.9)	106 (30.5)	63 (18.1)	104 (29.9)	26 (7.5)	55.5
I19. Using the app, I had many more opportunities to interact with my health care provider.	9 (2.6)	18 (5.2)	28 (8.1)	119 (34.3)	61 (17.6)	89 (25.6)	23 (6.6)	49.9
I20. I felt confident that any information I sent to my provider using the app would be received.	6 (1.7)	14 (4)	29 (8.3)	115 (33)	73 (21)	90 (25.9)	21 (6)	52.9
I21. I felt comfortable communicating with my health care provider using the app.	7 (2)	16 (4.6)	22 (6.3)	99 (28.3)	80 (22.9)	104 (29.7)	22 (6.3)	58.9

**Table 3:** Exploratory factor analysis results after Varimax rotation.

Item	Factor		
	System Information Arrangement	Usefulness	Ease of Use and Satisfaction
1	0.29	0.05	0.59
2	0.37	0.05	0.72
3	0.22	0.25	0.48
4	0.24	0.24	0.46
5	0.19	0.15	0.52
6	0.14	0.17	0.60
7	0.24	0.21	0.75
8	0.20	0.24	0.55
9	0.56	0.31	0.25
10	0.62	0.33	0.25
11	0.58	0.38	0.37
12	0.67	0.30	0.27
13	0.73	0.38	0.16
14	0.63	0.51	0.03
15	0.08	0.49	0.20
16	0.15	0.66	0.38
17	0.21	0.73	0.32
18	0.33	0.81	0.11
19	0.22	0.82	0.13
20	0.30	0.81	0.10
21	0.33	0.82	0.12
% Variance explained	24.9	23.7	19.2

Note. Factor loadings are presented in the table.

**Table 4:** Item-total correlation coefficients and the Cronbach’s Alpha coefficients if item deleted for MAUQ factors.

	Corrected Item-Total Correlation	Cronbach’s Alpha If Item Deleted	Cronbach’s Alpha
Ease of use and satisfaction			0.92
I1. The app was easy to use.	0.76	0.91	
I2. It was easy for me to learn to use the app.	0.69	0.91	
I3. I like the interface of the app.	0.77	0.91	
I4. The information in the app was well organized. so I could easily find the information I needed.	0.76	0.91	
I5. I feel comfortable using this app in social settings.	0.76	0.91	
I6. The amount of time involved in using this app has been fitting for me.	0.71	0.91	
I7. I would use this app again.	0.66	0.92	
I8. Overall. I am satisfied with this app.	0.80	0.91	
System information arrangement			0.89
I9. Whenever I made a mistake using the app. I could recover easily and quickly.	0.61	0.88	
I10. This mHealth app provided an acceptable way to receive health care services.	0.76	0.86	
I11. The app adequately acknowledged and provided information to let me know the progress of my action.	0.72	0.86	
I12. The navigation was consistent when moving between screens.	0.70	0.86	
I13. The interface of the app allowed me to use all the functions (such as entering information. responding to reminders. viewing information) offered by the app.	0.76	0.86	
I14. This app has all the functions and capabilities I expect it to have.	0.65	0.87	
Usefulness			0.92
I15. The app would be useful for my health and well-being.	0.56	0.92	
I16. The app improved my access to health care services.	0.71	0.91	
I17. The app helped me manage my health effectively.	0.79	0.90	
I18. The app made it convenient for me to communicate with my health care provider.	0.80	0.90	
I19. Using the app. I had many more opportunities to interact with my health care provider.	0.78	0.91	
I20. I felt confident that any information I sent to my provider using the app would be received.	0.79	0.90	
I21. I felt comfortable communicating with my health care provider using the app.	0.82	0.90	

**Table 5:** Factors’ descriptive measures and their intercorrelations.

		Minimum	Maximum	Mean (SD)	Median (IQR)	Spearman Correlation Coefficients (rho)			
						1.	2.	3.	4.
1.	Ease of use and satisfaction	1.25	7.00	5.66 (0.82)	5.88 (5.25–6)	1.00	0.74	0.58	0.85
2.	System information arrangement	1.33	7.00	5.17 (0.91)	5.33 (4.67–5.67)		1.00	0.70	0.90
3.	Usefulness	1.14	7.00	4.97 (1.02)	5.14 (4.29–5.71)			1.00	0.88
4.	Total MAUQ score	1.24	7.00	5.29 (0.82)	5.43 (4.90–5.81)				1.00

Note. All correlation coefficients were significant with *p* < 0.001.

**Table 6:** MAUQ factors by gender.

	Gender				P Mann–Whitney Test
	Men		Women		
	Mean (SD)	Median (IQR)	Mean (SD)	Median (IQR)	
Ease of use and satisfaction	5.59 (0.93)	5.75 (5–6.13)	5.7 (0.76)	5.94 (5.38–6)	0.303
System information arrangement	5.02 (1.04)	5.17 (4.33–5.67)	5.24 (0.83)	5.5 (4.83–5.83)	0.028
Usefulness	4.79 (1.15)	4.86 (4.14–5.57)	5.06 (0.94)	5.14 (4.43–5.86)	0.027
Total MAUQ score	5.16 (0.93)	5.33 (4.67–5.71)	5.35 (0.75)	5.57 (4.95–5.86)	0.028

## Data Availability

The raw data supporting the conclusions of this article will be made available by the authors on request.
